# Complicated Appendicitis in a Universal Healthcare System: Do Ethnic Disparities Persist?

**DOI:** 10.1007/s10728-025-00558-7

**Published:** 2026-01-30

**Authors:** Noam Weiner, Nitzan Goldberg, Eyal Meir, Ossama Abu-Hatoum, Uri Kaplan

**Affiliations:** 1 Department of General Surgery B, Emek Medical Center, Afula, Israel; 2 Department of General Surgery, Meir Medical Center, Kfar Saba, Israel; 3 Rappaport Faculty of Medicine, Technion Israel Institute of Technology, Technion City, Israel

**Keywords:** Ethnicity, Universal health coverage, Complicated appendicitis, Surgical delay, Elderly patients

## Abstract

While ethnic disparities in appendicitis outcomes have been previously documented, limited data exist regarding their influence on the incidence of complicated appendicitis within population covered by universal health insurance. This study aimed to assess whether ethnicity (Israeli Jewes vs. Israeli Arabs) is independntly accosioated with the risk of complicated appendicitis in the context of Israel’s universal healthcare system. All adult patients who underwent appendectomy at our institution between January 2010 and December 2021. The primary outcome was appendicitis severity, categorized as complicated or uncomplicated based on pathology reports. Secondary outcomes included length of hospital stay and rehospitalization within six months. Univariable and multivariable logistic regression analyses were performed to identify factors associated with complicated appendicitis. A total of 2,943 patients were included. In the multivariable logistic regression analysis, prolonged in-hospital delay before surgery exceeding 12 h increased the risk of complicated appendicitis (OR = 1.41; 95% CI: 1.14–1.75; *p* = 0.002). Age over 60 years doubled the risk of complicated appendicitis compared to younger patients (OR = 2.42; 95% CI: 1.81–3.24; *p* < 0.001). Although Jewish patients were older and had higher rates of complicated appendicitis, ethnicity was not independently associated with complicated appendicitis after adjustment for age, comorbidities, and surgical delay. Ethnicity was not an independent risk factor for complicated appendicitis in a population with universal health coverage. Older age and prolonged in-hospital delay were the primary predictors of complicated disease.

## Background

Acute appendicitis is among the most common emergencies in general surgery, with a lifetime incidence estimated at 7–8% [1]. Simple appendicitis is typically characterized by uncomplicated or phlegmonous inflammation, whereas complicated appendicitis (CA) involves appendiceal necrosis, perforation or the developement of peri-appendicular abscess. CA accounts for approximatly 16% and 30% of all cases [2]. Well-established risk factors for CA include extremes of age, with young children [3, 4] and older adults [4, 5] particularly susceptible. Additional contributors include residence in rural areas [6–8], and delayed presentation or diagnosis [9].

Previous studies investigation the incidence of appendiceal perforation among ethnic minorities have yielded inconsistent findings [7, 10–13]. Notably, several of these studies included populations with differing level of health insurance coverage, which may have confounded the relationship between ethnicity and disease severity.

Israel is a multiethnic society comprising several distinct population groups, primarily including Jewish (approximately 74%) and Arab (approximately 21%) populations, alongside smaller communities such as Druze and other minorities. These groups differ in cultural practices, health behaviors, socioeconomic status, and patterns of healthcare utilization, all of which may contribute to disparities in disease prevalence, severity, and outcomes.

Studies have reported higher rates of chronic conditions such as diabetes, obesity, and cardiovascular disease among Israeli Arabs (IA) compared to Israeli Jews (IJ), often attributed to lower socioeconomic status, reduced access to preventive care, and differing health literacy levels [14–17]. Temporal factors were reported in the literature as a prognostic factors for the development of CA. There’s an increace incident of acute appendicitis during warmer weather [18, 19]. Most patients with acute appendicitis presented in the afternoon/evening however, patients with CA presented during workday [20]. In hospital delay of surgery (> 8 h) was associated with higer risk for CA [21].

Israel maintains a universal healthcare system characterized by mandatory health insurance and equitable access to medical services for all citizens. Israeli citizens receive medical treatment based on medical insurance provided by four health care providers which subsidize medical treatment such as primary care, hospitalizations and emergency surgical procedures. Clalit Health Services (CHS) is the largest healthcare provider in northern Israel, providing health coverage for more than 50% of the population. Our institution functions as a central referral center for CHS beneficiaries.

Despite the provision of universal health coverage, substantial socioeconomic disparities persist between IA and IJ populations [17]. This distinct healthcare environment, which ensures equitable access to emergency medical services while encompassing notable socioeconomic heterogeneity, presents a unique opportunity to investigate whether ethnic disparities in the incidence of CA endure under conditions of equal healthcare accessibility.

The primary objective of our study was to assess whether ethnicity (IJ vs. IA) is independently associated with the risk of complicated appendecitis.

## Methods

### Ethics

This retrospective cohort study was conducted at Emek Medical Center following approval from the institutional Helsinki Committee (Approval number: 0092-21-EMC). The study was conducted in accordance with ethical guidelines governing research involving human subjects. Due to its retrospective design and the utilization of anonymized data, the requirement for informed consent was waived by the ethics committee. Confidentiality of participant data was strictly maintained, with all identifying information accessible exclusively to the research team.

## Population and Patient Selection

**Fig. 1:**
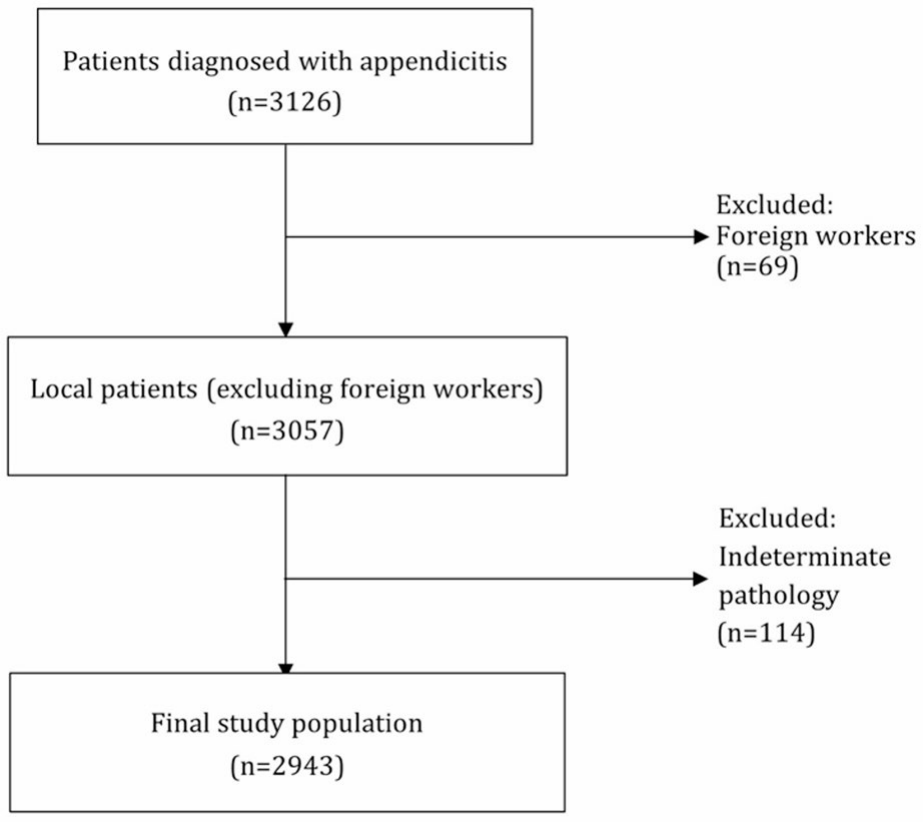
Flowchart illustrating patient screening and selection. A total of 3126 patients diagnosed with appendicitis who underwent surgery between 2000 and 2021 were assessed for eligibility. Sixty-nine foreign workers were excluded from the analysis. An additional 114 patients were excluded due to indeterminate pathological classification (unable to differentiate between uncomplicated and complicated appendicitis). The final study population included 2,943 local patients

The study population comprised all adult patients (≥ 18 years of age) who underwent appendectomy at Emek Medical Center between January 2010 and December 2020. Exclusion criteria included pregnant patients, individuals unable to provide a reliable medical history, appendiceal neoplasm and cases with incomplete medical records - particularly those lacking essential data such as histopathological finding of the appendix. The process of patient screening and selection is illustrated in Fig. 1.

## Data Collection

### Baseline Characteristics and Demographics

Data were retrospectively extracted from electronic medical records and included both demographic and clinical characteristics. Demographic data comprised age, sex, ethnicity, and place of residence.residential location was categorized according to settlement size, based on Israeli population data, as follows: less than 5,000 residents, 5,000 to 20,000 residents, 20,000 to 100,000 residents, and more than 100,000 residents. Documented comorbidities included diabetes mellitus, hypertension, hyperlipidemia, ischemic heart disease, and obstructive sleep apnea.

## Temporal Factors

Temporal variables related to hospital presentation were assessed to explore potential association with disease severity and clinical outcomes. The season of presentation was categorized by calendar months as follows: winter (January–March), spring (April–June), summer (July–September), and fall (October–December). Hospital shift at time of presentation was defined based on arrival time at the emergency department: morning shift (07:00–15:00), evening shift (15:00–23:00), and night shift (23:00–07:00). Additionally, arrival times were dichotomized into daytime (07:00–19:00) and nighttime (19:00–07:00) intervals.

Time to surgery was defined as the interval between emergency department (ED) registration and the initiation of the surgical procedure. At our institution, the primary treatment for acute appendicitis across all attending surgeons is laparoscopic appendectomy. Patients presenting with a periappendiceal abscess or those who decline surgical intervention are managed conservatively with antibiotic therapy. Perioperative antibiotic prophylaxis consists of three doses administered around the time of surgery. Hospital length of stay was calculated from the time of ED arrival to the time of hospital discharge.

### Primary and Secondary Outcomes

The primary outcome was the appendicitis severity, as determined by the final pathological diagnosis. Cases exhibiting evidence of perforation, abscess, or tissue necrosis were classified as complicated appendicitis (CA), whereas cases characterized by inflammation or phlegmon were categorized as simple appendicitis.

Secondary outcomes included the length of hospital stay and readmissions within six months.

### Statistical Analysis

Univariable analyses were conducted to compare baseline characteristics and clinical outcomes between patients with complicated and simple appendicitis. Categorical variables were analyzed using the Chi-square test, while continuous variables were evaluated using independent samples t-tests. Variables demonstrating statistical significance in univariable analyses were subsequently entered into multivariable logistic regression models to identify factors independently associated with complicated appendicitis.

A secondary stratified analysis was performed by dividing the cohort into three age groups: < 30 years, 30–60 years, and > 60 years. Univariable and multivariable analyses were repeated within each age stratum to assess age-specific associations between baseline variables and clinical outcomes.

All statistical analyses were conducted using IBM SPSS Statistics, version 25.0 (IBM Corp., Armonk, NY, USA). A two sided p-value of < 0.05 was considered indicative of statistical significance.

## Results

We identified 2,943 patients aged 18 years old or older, admitted to our institution with the diagnosis of acute appendicitis between January ^st^, 2010, and December 31th, 2021. The mean age was 37 years (range: 18–99), and 58% of the cohort were males (*n* = 1,694). Comorbidities were relatively uncommon and heterogenous, with hypertension and hyperlipidemia being the most prevalent, observed in 13.3% (*n* = 391) and 12.6% (*n* = 371), respectively. Among the entire cohort, 78% (*n* = 2,296) were diagnosed with simple appendicitis, while 22% (*n* = 647) were classified as having CA.

The comparative characteristics of patients with complicated versus simple appendicitis are summarized in Table 1. Patients diagnosed with CA were significantly older, had a greater burden of comorbidities, and more frequently presented during morning shifts. Additionally, these patients experienced a longer interval from ED arrival to surgical intervention, longer hospital stay, and higher rate of rehospitalization within six months, compared to patients with simple appendicitis.

**Table 1:** Descriptive baseline characteristics of patients with simple and complicated appendicitis

	Severity of inflammatory process	
Variable	Simple	Complicated
N (%)	2296 (78)	647 (22)
Age (years)	34.1 ± 14.4	46.6 ± 19.2*
Gender		
Female	970 (42.2)	279 (43.1)
Male	1326 (57.8)	368 (56.9)
Settlement size distribution		
< 5000	533 (23.5)	158 (25.0)
5001-20,000	619 (27.3)	163 (25.8)
20,001–100,000	1063 (46.8)	296 (46.9)
> 100,000	55 (2.4)	14 (2.2)
Small vs. large town classification		
< 20,000	1152 (50.7)	321 (50.9)
> 20,000	1118 (49.3)	310 (49.1)
Comorbidities		
Diabetes Mellitus	34 (1.5)	38 (5.9)
Ischemic Heart Disease	232 (10.1)	159 (24.6)
Hyperlipidemia	226 (9.8)	145 (22.4)
Hypertension	119 (5.2)	106 (16.4)
Obstructive sleep apnea	14 (0.6)	10 (1.5)
Season of admission		
Winter	527 (23.0)	136 (21.0)
Spring	566 (24.7)	171 (26.4)
Summer	621 (27.0)	186 (28.7)
Autumn	582 (25.3)	154 (23.8)
ED arrivals distribution over 24 h		
Arrivals by shift		
Morning (7:00–16:00)	1026 (44.7)	349 (53.9)*
Evening (16:00–24:00)	968 (42.2)	233 (36.0)*
Night (24:00–7:00)	302 (13.2)	65 (10.0)*
Day vs. Night hours arrivals		
Day	1408 (61.3)	427 (66)*
Night	888 (38.7)	220 (34)*
Time from ED arrival to surgery (hours)	11.12 ± 10.58	19.41 ± 33.03
Length of hospitalization (days)	2.22 ± 0.95	8.01 ± 6.79*
Re-hospitalization within 6 months	101 (4.4)	122 (18.9)*

*ED* Emergency department

*P < 0.05 vs. simple, independent samples t-test or Chi-square test, as appropriate

In the multivariable logistic regression model, a prolonged interval from ED arrival to surgery remained an independent predictor of CA. When analyzed as a continuous variable, each additional hour of delay was associated with a 3% increase in the odds of developing CA (OR = 1.03; 95% CI: 1.02–1.04; *p* < 0.001). Furthermore, surgical delays exceeding 12 h were independently associated with increased odds of CA (OR = 1.41; 95% CI: 1.14–1.75; *p* = 0.002). No significant associations were observed for gender, ethnicity, any of the comorbidities, or settlement size after adjustment for age and surgical delay.

**Fig. 2:**
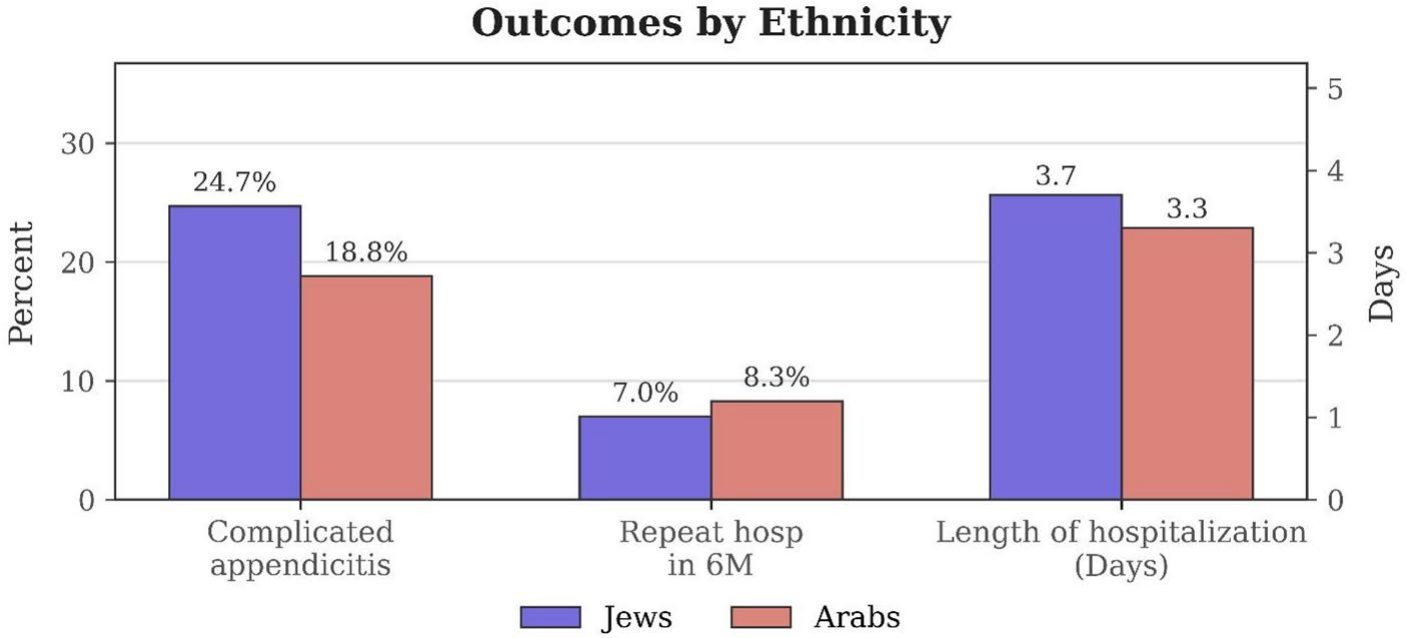
Comparison of appendicitis severity, repeat hospitalization, and hospital stay by ethnicity. Data are presented as percentages (%) for categorical variables and mean values (in days) for continuous variables. Categorical variables include the rate of complicated appendicitis and repeat hospitalization. The rate of complicated appendicitis was significantly higher among Jewish patients compared to Arab patients (24.7% vs. 18.8%, Chi-square test, *p* = 0.00016). Repeat hospitalization rates were slightly higher in Arab patients (8.3%) than in Jewish patients (7.0%), but this difference was not statistically significant (Chi-square test, *p* = 0.175). The mean length of hospital stay was longer in Jewish patients (3.7 days) compared to Arab patients (3.3 days), with a statistically significant difference (Kruskal-Wallis test, *p* = 0.015)

Baseline characteristics and temporal factors differed significantly between IJ and IA patients, as summarized in Table 2. IJ patients presented at an older age, had higher rates of hypertension (while other comorbidities were comparable), were more likely to reside in urban centers, and more frequently arrived at the hospital during morning shifts compared to IA patients. Furthermore, IJ patients had a higher incidence of CA and longer hospital stays. However, rates of repeat hospitalization were comparable between the groups (Fig. 2).

**Table 2:** Baseline characteristics and temporal factors of presentation of Jews and Arabs

	Ethnicity	
Variable	Jews	Arab
Sample size	1593 (54.1)	1350 (45.9)
Age (Years)	39.7 ± 17.7)	33.4 ± 14.0*
Gender		
Female	729 (45.8)	520 (38.5)*
Male	864 (54.2)	830 (61.5)*
Settlement size distribution		
< 5000	506 (32.6)	185 (13.7)*
5001–20,000	185 (11.9)	597 (44.3)*
20,001–100,000	792 (51.0)	567 (42.0)*
> 100,000	69 (4.4)	0 (0.0)*
Small vs. Large town classification		
< 20,000	691 (44.5)	782 (58.0)*
> 20,000	861 (55.5)	567 (42.0)*
Comorbidities		
Diabetes mellitus	32 (2.0)	40 (3.0)
Ischemic heart disease	125 (7.8)	100 (7.4)
Hyperlipidemia	218 (13.7)	153 (11.3)
Hypertension	234 (14.7)	157 (11.6)*
Obstructive sleep apnea	12 (0.8)	12 (0.9)
Season of admission		
Winter	367 (23.0)	296 (21.9)
Spring	395 (24.8)	342 (25.3)
Summer	429 (26.9)	378 (28.0)
Autumn	402 (25.2)	334 (24.7)
ED arrivals distribution over 24 h		
Arrivals by shift		
Morning (7:00–16:00)	774 (48.6)	601 (44.5)*
Evening (16:00–2400)	644 (40.4)	557 (41.3)*
Night (24:00–7:00)	175 (11.0)	192 (14.2)*
Day vs. Night hours arrivals		
Day	1042 (65.4)	793 (58.7)*
Night	551 (34.6)	557 (41.3)*

*ED* Emergency department

*P < 0.05 vs. Jews, independent samples t-test or Chi-square test, as appropriate

Baseline demographic and clinical characteristics, comorbidities, and hospital presentation patterns also varied significantly across age groups, as presented in Table 3. Older patients demonstrated a distinct ethnic distribution, were more likely to be IJ and a higer prevalence of comorbidities, including diabetes mellitus, ischemic heart disease, hyperlipidemia, and hypertension. They were also more likely to reside in small towns, present during daytime hours, and experience longer delays from ED arrival to surgical intervention.

**Table 3:** Baseline characteristics and Temporal factors of presentation across age groups

	Age groups (years)		
Variable	0–30	30–60	60<
Sample size(%)	1353 (46)	1261 (43)	257 (11)
Ethnicity			
Jews(%)	642 (47.5)	692 (54.9)	257 (78.6)*
Arabs(%)	711(52.5)	569 (45.1)	70 (21.4)*
Comorbidities			
Diabetes mellitus(%)	2 (0.1)	32 (2.5)	38 (11.6)*
Ischemic heart disease(%)	24 (1.8)	82 (6.5)	119 (36.4)*
Hyperlipidemia (%)	3 (0.2)	189 (15.0)	178 (54.4)*
Hypertension(%)	20 (1.5)	169 (13.4)	201 (61.5)*
Obstructive sleep apnea(%)	6 (0.4)	9 (0.7)	9 (2.8)*
Small vs. Large town classification			
< 20,000(%)	630 (47.1)	669 (54.1)	174 (53.2)*
> 20,000(%)	707 (52.9)	567(45.9)	153 (46.8)*
ED arrivals distribution over 24 h			
Day (%)	785 (58.0)	829 (65.7)	220 (67.3)*
Night (%)	568 (42.0)	432 (34.3)	107 (32.7)*
ED arrival to surgery (hours)(mean ± SD)	11.71 ± 15.6	12.58 ± 17.30	17.69 ± 24.5*

*ED* Emergency Department

*P < 0.05 vs. other age groups, Kruskal-Wallis or Chi-square test, as appropriate

**Fig. 3:**
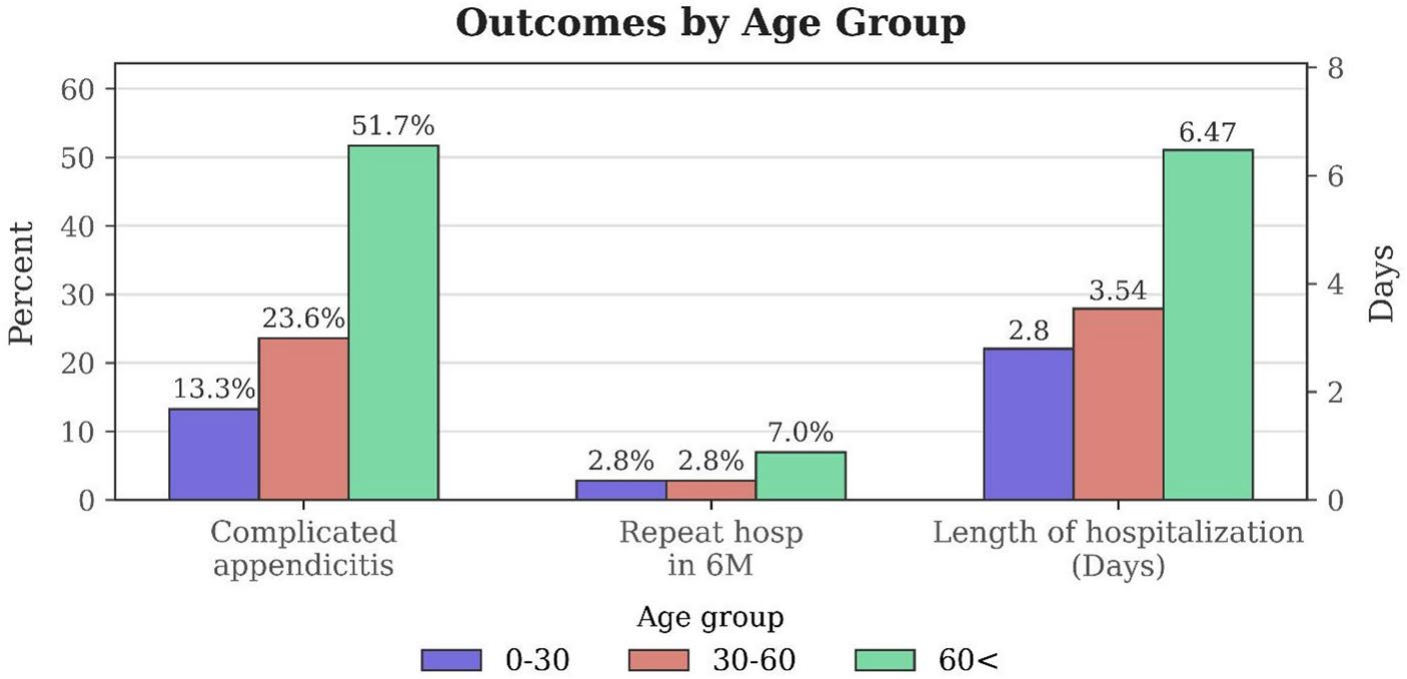
Comparison of appendicitis severity, repeat hospitalization, and hospital stay across age groups. Data are presented as percentages (%) for categorical variables and mean days for continuous variables. The rate of complicated appendicitis and repeat hospitalization was significantly higher in older age groups (Chi-square test, *p* < 0·001). The length of hospitalization increased with age and was compared across groups using the Kruskal-Wallis test, demonstrating a statistically significant difference (*p* < 0·001)

Clinical outcomes worsened progressively with increasing age (Fig. 3). The rate of CA rose sharply with age: patients over 60 years of age had more than twice the rate observed in those aged 30–60, and nearly four times the rate found in patients under 30 years. Similarly, hospital length of stay increased with age. Patients over 60 had hospitalizations nearly twice as long as those aged 30–60, and more than double the duration compared to patients under 30 years. Repeat hospitalizations within six months also occurred more frequently in the oldest age group, with patients over 60 experiencing more than double the re-hospitalization rate compared to both younger groups.

In multivariable logistic regression analysis, age greater than 60 years emerged as an independent predictor of CA (OR = 2.42, 95% CI: 1.81–3.24, *p* < 0.001). In a separate model in which age was treated as a continuous variable, each additional year of age was associated with a 3% increase in the odds of complicated appendicitis (OR = 1.03, 95% CI: 1.02–1.04, *p* < 0.001).

## Discussion

This study evaluated whether IA patients residing in northern Israel exhibit an increased risk of developing CA compared to their IJ patients counterparts, within the contex of a universal healthcare system designed to provide equitable access to emergency surgical services. The retrospective analysis of our cohort did not identify an association between IA ethnicity and the risk of CA. The unadjusted rates of CA were higher among IJ patients, however, multivariable logistic regression analysis adjusted for age and comorbidities revealed that ethnicity was not an independent risk factor (OR = 0.939, 95% CI 0.774–1.139; *P* = 0.523). The observed disparity in complicated disease appears to be primarily attributable to age differences at presentation.

Our study supports the hypothesis that equal access to medical treatment diminishes ethnic differences in outcomes. A prior study found racial and ethnic disparities in delayed diagnosis of appendicitis in children [22]; however, another publication evaluating the effect of race and socioeconomic status on appendicitis outcomes in a setting with equal healthcare access found that these factors did not influence disease severity or length of hospitalization [11]. Notably, a recent Israeli study reported that foreign workers exhibited significantly higher adjusted odds ratio for CA compared to the general population [23]. The authors proposed that disease severity in this group may be influenced by behavioral factors and work-related concerns that delay care-seeking.

The association between advanced age and disease severity in appendicitis is well established in the literature [5, 24]. In the present study, this relationship was corroborated by two key findings. First, the mean age of IJ patients was significantly higher than that of IA patients (39.7 years versus 33.4 years, respectively (*P* < 0.001)). Second, age stratification analysis demonstrated a clear, progressive increase in the incidence of CA with advancing age, with rates exceeding 50% among patients aged over 60 years. Notably, IJ patients constituted nearly 80% of this older age cohort, compared to 55% of those aged 30–60-year group and 47% of those under 30 years. This age-related demographic distribution likely accounts for a substantial portion of the observed ethnic disparity in disease severity.

Secondary outcomes supported the primary findings. Ethnicity had only a marginal effect on length of hospitalization, while age had a substantial impact. Although the difference in length of stay between IJ and IA patients was statistically significant, it amounted to only ~ 10 h and was unlikely to be clinically meaningful. In contrast, patients aged over 60 years were hospitalized for approximately three days longer than those aged 30–60 years, and nearly four days longer than patients under 30 years. Similarly, age- but not ethnicity- was associated with an increased rate of rehospitalization, with older patients demonstrating a higher likelihood of readmission than younger cohorts.

Although the IA group in our study represents a population with relatively lower socioeconomic and educational attainment compared to the IJ group [17], the presence of a comprehensive national health insurance system that guarantees access to emergency medical services may mitigates the typical barriers associated with socioeconomic disadvantage. These findings align with previous studies demonstrating that when access to medical care is equitable, disparities in clinical outcomes can be substantially attenuated [11, 13, 25]. Our results reinforce this perspective, highlighting the protective role of a universal healthcare framework in reducing ethnic disparities in acute surgical conditions.

Our data also indicate that both advanced age and in-hospital delay to surgery are significant predictors of CA. After adjusting for comorbidities, ethnicity, and time to surgery, age > 60 years remained significantly associated with CA (OR = 2.42, 95% CI: 1.81–3.24, *P* < 0.001). Likewise, delays exceeding 12 h from ED arrival to surgery were associated with increased odds of complicated appendicitis (OR = 1.41, 95% CI: 1.14–1.75, *P* = 0.002), independent of age, gender, comorbidities, and ethnicity. These findings corroborate previous reports identifying advanced age and delayed intervention as key risk factors for perforated appendicitis [4, 5, 26, 27].

Patients aged over 60 years in our cohort experienced longer in-hospital delays prior to surgery compared to younger patients. This delay may be multifactorial. Older patients often present with non-specific symptoms such as diffuse abdominal pain, general malaise, or fever, necessitating extended diagnostic workups. Unlike younger patients who are frequently evaluated using ultrasonography, older individuals typically require computed tomography (CT) imaging due to a broader differential diagnosis [28]. Notably, during the earlier years of the study period, abdominal CT protocols included oral contrast administration, necessitating a waiting period of approximately three hours. Moreover, elderly patients more frequently require preoperative medical optimization due to a higher burden of comorbidities.

Overall, these findings suggest that in the context of universal health coverage, ethnicity is not an independent risk factor for CA. This suggests that disparities reported in other settings may be largely attributable to differences in healthcare access rather than intrinsic ethnic or socioeconomic variables. Importantly, age and in-hospital surgical delay emerged as the principal determinants of disease severity. Elderly patients, who also experienced longer hospital stays and higher rehospitalization rates, should be prioritized for expedited diagnostic and surgical intervention. Addressing delays in this high-risk population may improve outcomes and reduce healthcare resource utilization.

This study has several limitations inherent to its retrospective design. First, reliance on electronic medical records may introduce information bias due to incomplete or inaccurate documentation. For example, due to limitations in extracting reliable and consistent data on symptom onset from our electronic medical records, we were unable to accurately determine time to presentation. For this reason, we elected to exclude this variable from the analysis. Second, although we adjusted for major demographics and clinical variables, residual confounding from unmeasured factors such as individual-level socioeconomic status or health literacy may persist. Third, categorization into broad ethnic groups (Jewish vs. Arab) may overlook significant heterogeneity within each group. Fourth, the 21-year study period encompassed changes in imaging protocols and surgical techniques, including the adoption of laparoscopic appendectomy as the standard of care, potentially affecting the timing and approach to treatment. Fifth, the single-center setting in northern Israel may limit the generalizability of these findings to other geographic regions or healthcare environments. Lastly, the observational design precludes causal inference between identified predictors and clinical outcomes.

## Conclusion

In this large retrospective study conducted in a region with universal healthcare access, we found no evidence that IA ethnicity independently predicts CA. Instead, advanced age and in-hospital delay were the primary determinants of disease severity. These findings underscore the importance of timely diagnosis and intervention—particularly in elderly patients—to reduce complication rates and optimize outcomes.

## Data Availability

The data that support the findings of this study are not openly available due to reasons of sensitivity and are available from the corresponding author upon reasonable request. Data are in controlled access data storage at Clalit Health Services database.
